# Increased Susceptibility to Cerebral Microhemorrhages Is Associated With Imaging Signs of Microvascular Degeneration in the Retina in an Insulin-Like Growth Factor 1 Deficient Mouse Model of Accelerated Aging

**DOI:** 10.3389/fnagi.2022.788296

**Published:** 2022-03-09

**Authors:** Lauren R. Miller, Stefano Tarantini, Ádám Nyúl-Tóth, Morgan P. Johnston, Teryn Martin, Elizabeth C. Bullen, Marisa A. Bickel, William E. Sonntag, Andriy Yabluchanskiy, Anna Csiszar, Zoltan I. Ungvari, Michael H. Elliott, Shannon M. Conley

**Affiliations:** ^1^Department of Cell Biology, University of Oklahoma Health Sciences Center, Oklahoma City, OK, United States; ^2^Vascular Cognitive Impairment and Neurodegeneration Program, Oklahoma Center for Geroscience and Healthy Brain Aging, Department of Biochemistry and Molecular Biology, University of Oklahoma Health Sciences Center, Oklahoma City, OK, United States; ^3^Stephenson Cancer Center, University of Oklahoma, Oklahoma City, OK, United States; ^4^International Training Program in Geroscience, Doctoral School of Basic and Translational Medicine/Department of Public Health, Semmelweis University, Budapest, Hungary; ^5^International Training Program in Geroscience, Institute of Biophysics, Biological Research Centre, Eötvös Loránd Research Network, Szeged, Hungary; ^6^Department of Ophthalmology, Dean McGee Eye Institute, University of Oklahoma Health Sciences Center, Oklahoma City, OK, United States; ^7^Department of Physiology, University of Oklahoma Health Sciences Center, Oklahoma City, OK, United States

**Keywords:** insulin-like growth factor 1, retina, intracerebral hemorrhage, retinal vasculature, microhemorrhage, aging, cerebrovascular aging, vascular cognitive impairment

## Abstract

Age-related cerebrovascular defects contribute to vascular cognitive impairment and dementia (VCID) as well as other forms of dementia. There has been great interest in developing biomarkers and other tools for studying cerebrovascular disease using more easily accessible tissues outside the brain such as the retina. Decreased circulating insulin-like growth factor 1 (IGF-1) levels in aging are thought to contribute to the development of cerebrovascular impairment, a hypothesis that has been supported by the use of IGF-1 deficient animal models. Here we evaluate vascular and other retinal phenotypes in animals with circulating IGF-1 deficiency and ask whether the retina mimics common age-related vascular changes in the brain such as the development of microhemorrhages. Using a hypertension-induced model, we confirm that IGF-1 deficient mice exhibited worsened microhemorrhages than controls. The retinas of IGF-1 deficient animals do not exhibit microhemorrhages but do exhibit signs of vascular damage and retinal stress such as patterns of vascular constriction and Müller cell activation. These signs of retinal stress are not accompanied by retinal degeneration or impaired neuronal function. These data suggest that the role of IGF-1 in the retina is complex, and while IGF-1 deficiency leads to vascular defects in both the brain and the retina, not all brain pathologies are evident in the retina.

## Introduction

Aging elicits multifaceted functional and structural impairment in the cerebral microcirculation, which has a critical role in the pathogenesis of vascular cognitive impairment and dementia (VCID) (Hajdu et al., 1990; Mayhan et al., 1990; Zlokovic, 2011; Jessen et al., 2015; Cooper et al., 2016; Daulatzai, 2016; Ighodaro et al., 2016). Within the spectrum of age-related microvascular pathologies [including microvascular rarefaction (Tarantini et al., 2016; Toth et al., 2017; Norling et al., 2020; Nyul-Toth et al., 2021), disruption of the blood–brain barrier (BBB) (Verheggen et al., 2020; Nyul-Toth et al., 2021), impaired regulation of cerebral blood flow (CBF) (Tarantini et al., 2017b,^2021^), amyloid pathologies], recent studies highlighted the pathogenic role of cerebral microhemorrhages (CMHs) in the genesis of VCID (Poels et al., 2011, 2012; Ungvari et al., 2017). CMHs are small (<5 mm) bleeds that develop due to the rupture of cerebral arterioles and capillaries. Aging significantly increases microvascular fragility (in part due to microvascular degeneration), which exacerbates the genesis of CMHs, and CMH burden predicts cognitive decline in older adults (Kato et al., 2002; Vernooij et al., 2008; Ungvari et al., 2017).

Despite its pathophysiological importance, the human cerebral microcirculation is not readily accessible for imaging and functional assessment. The desire to identify novel, accessible biomarkers and tools for interrogating the cerebromicrovascular changes that contribute to VCID has led to much work utilizing the retinal vasculature as a proxy for the brain vasculature (Lipecz et al., 2019; McGrory et al., 2019; Czako et al., 2020). Cerebral and retinal vasculature share developmental and anatomical origins. The retina is considered part of the central nervous system, developing from the diencephalon, and exhibits similar anatomical features and physiological properties, including microvascular architecture, energy requirements, regulation of blood flow, and vascular barrier function (London et al., 2013; Tata et al., 2015; Lipecz et al., 2019). There is increasing evidence that age-related pathophysiological processes that affect the central nervous system and the cerebral microcirculation have a direct profound impact on the retina and retinal microcirculation as well (Stanton et al., 1995; Liew et al., 2008; Che Azemin et al., 2013; Csipo et al., 2019; Lipecz et al., 2019). Yet it is not clear whether the retina exhibits all of the tissue level pathologies that are associated with cerebrovascular aging. Importantly, the association between increases in microvascular fragility in the brain and the retina has not yet been investigated.

The mechanisms by which aging exacerbates functional and structural impairment in the cerebral microcirculation and promotes the genesis of CMHs include an age-related decline in circulating insulin-like growth factor 1 (IGF-1) (Sonntag et al., 2013; Toth et al., 2014, 2015a; Ashpole et al., 2017; Tarantini et al., 2017c,^2021^; Fulop et al., 2018). IGF-1 is a vasoprotective growth factor largely produced by the liver, whose circulating levels significantly decrease with increasing age (Ungvari and Csiszar, 2012; Sonntag et al., 2013; Tarantini et al., 2017c). Animal models of circulating IGF-1 deficiency serve as models of accelerated aging, mimicking many age-related cerebrovascular pathologies. These include impaired myogenic autoregulation, impaired neurovascular coupling, microvascular rarefaction, decreased cerebral blood flow, and consequential impairment of higher brain functions (Bailey-Downs et al., 2012; Sonntag et al., 2013; Toth et al., 2014, 2015a; Fulop et al., 2018). Decreased circulating IGF-1 levels in rodent models are also associated with increased BBB permeability, pathological microvascular remodeling, increased microvascular fragility, and increased susceptibility to the hypertension-induced development of CMHs (Bake et al., 2014, 2016; Tarantini et al., 2017c). Yet, there are no studies extant investigating the role of circulating IGF-1 deficiency in microvascular pathologies in the retina.

The present study was designed to test the hypothesis that adult-onset circulating IGF-1 deficiency promotes the development of a pro-fragility microvascular phenotype both in the brain and the retina. To test our hypothesis, we used an established murine model of isolated endocrine IGF-1 deficiency: knockdown of IGF-1 specifically in the liver using Cre-lox technology (*Igf1^f/f^* + TBG-Cre-AAV8) (Tarantini et al., 2017c). To test microvascular fragility, we induced chronic hypertension in IGF-1 deficient mice and respective controls [by treatment with angiotensin II (Ang II) and L-NAME, a NO synthase inhibitor (Liu et al., 1998; Ho et al., 2008; Bailey-Downs et al., 2012; Toth et al., 2013, 2015a; Tarantini et al., 2017c)] and assessed occurrence of microhemorrhages in both the brain and retina. We also assessed the effects of circulating IGF-1 deficiency on the structural integrity of the retina and the retinal vessels.

## Materials and Methods

### Experimental Animals

All animal work was reviewed and approved by the local Institutional Animal Care and Use Committee (IACUC; University of Oklahoma Health Sciences Center, Oklahoma City, OK, United States). Mice on the C57BL/6 background that were homozygous for an *Igf1* floxed allele (*Igf1*^f/f^*)* were purchased from Jackson Laboratories (line 016831). These mice have exon 4 of the *Igf1* gene flanked by loxP sites, allowing for the excision of this entire exon via Cre recombinase. Transcripts of the altered Igf1 gene yield a protein that fails to bind the IGF-1 receptor. IGF-1 deficiency was induced in *Igf1^f/f^* mice by adeno-associated virus (AAV8)-mediated expression of Cre recombinase in the liver at 4 months of age, as previously reported (Bailey-Downs et al., 2012; Toth et al., 2014). The AAV8 vector was acquired from the University of Pennsylvania Viral Vector Core (Penn Vector Core, Philadelphia, PA, United States^1^). The thyroxine-binding globulin (TBG) promoter was used to restrict the expression of the AAV8 vector to hepatocytes. At 4 months of age, *Igf1^f/f^* mice were randomly assigned to two groups and were administered approximately 1.3 × 10^10^ viral particles of AAV8-TBG-Cre or AAV8-TBG-eGFP via retro-orbital injection, as described (Tarantini et al., 2017c). The majority of circulating IGF-1 is produced in the liver. Since IGF-1 is critical for the development of many organ systems during adolescence, including the cardiovascular system, this model was used to specifically study the effects of adult-onset circulating IGF-1 deficiency (Toth et al., 2014). Animals were used for experiments at either approximately 1 year of age (12–14 months) or 2 years of age (24–27 months). Both male and female mice were used, however studies were not powered to evaluate the role of sex as a biological variable. Animals were housed in the Rodent Barrier Facility at OUHSC under specific pathogen-free barrier conditions, on a 12-h light/12-h dark cycle, with access to standard rodent chow (Purina Mills, Richmond, IN, United States) and water *ad libitum*. In-cage light levels during the light cycle were ∼30 lux.

### Induction of Hypertension

To assess microvascular fragility, hypertension was induced in study animals at 12–14 months of age. AAV8-TBG-Cre and AAV8-TBG-eGFP mice were randomly assigned to either the “hypertensive” (HT) or “normotensive” (NT) groups. Hypertension was induced by a combination treatment with ω-nitro-_*L*_-arginine-methyl ether [L-NAME (N5751, Millipore Sigma, St. Louis, MO, United States), 100 mg kg^–1^ day^–1^, in drinking water] and administration of angiotensin II [Ang II; s.c. via osmotic mini-pumps (Alzet Model 2006, 0.15 μL h^–1^, 42 days; Durect Co, Cupertino, CA, United States)]. Pumps were filled with either saline or a solution of angiotensin II (Sigma Chemical Co., St. Louis, MO, United States) that delivered 1 μg min^–1^ kg^–1^ of angiotensin II for up to 28 days. Mini-pumps were surgically placed in isoflurane anesthetized mice in the subcutaneous space on the back of the animal. This was accomplished by making a small incision in the intrascapular region, blunt dissection of the subcutaneous space, and closure of the incision with surgical sutures using aseptic techniques. Animals were given an s.c. injection of sustained release Buprenorphine (ZooPharm, Fort Collins, CO, United States) to manage post-operative pain. Animal blood pressure was measured via the tail-cuff method using the CODA Non-Invasive Blood Pressure System (Kent Scientific Co., Torrington, CT, United States) as described (Toth et al., 2015b). Mice were placed in a restrainer on an animal warmer for the duration of the measurement to encourage tail vein dilation for accurate blood pressure measurements.

### Standardized Neurological Examination

Mice underwent daily neurological examination to predict the presence of clinically manifest hemorrhages. The scoring system evaluates spontaneous activity, symmetry in limb movement, forelimb outstretching, climbing ability, body proprioception, and response to vibrissae touch. Scores were summed on an 18-point scale as a measure of neurological function. A decline in this neurological score correlates with cerebral hemorrhage development (Toth et al., 2015b). Mice were euthanized at a neurological score of 15 or lower, or when they reached day 28 post-hypertension surgery, whichever came first. Mice were euthanized via transcardial perfusion with ice-cold 1× phosphate buffered saline (1× PBS, 137 mM NaCl, 2.7 mM KCl 10 mM Na_2_HPO_4_, 1.8 mM KH_2_PO_4_, pH 7.4, pH 7.2) for 10 min under ketamine/xylazine (84/14 mg kg^–1^) anesthesia.

### Fundoscopy and Fluorescein Angiography

To assess vascular damage in the retina, fundus imaging and fluorescein angiography were performed using the Micron III system (Phoenix Research Laboratories, Pleasanton, CA, United States) as described (Koirala et al., 2013; Chakraborty et al., 2020). Mice were anesthetized with ketamine/xylazine (84, 7 mg kg^–1^)) and eyes were dilated with 1% cyclopentolate eye drops (Family Medicine Pharmacy, University of Oklahoma Health Sciences Center, Oklahoma City, OK, United States). One drop of 2.5% Gonak (McKesson Medical Surgical, Richmond, VA, United States) was applied to each eye. Bright field images were collected, and then animals were injected intraperitoneally with 100 μL of 1% (w/v) fluorescein sodium (Sigma-Aldrich, St. Louis, MO, United States) for angiography. Angiogram images were captured using a GFP filter. All fundus images were captured using StreamPix software (Phoenix Research Labs, Bend, OR, United States). We observed repeated patterns of constriction in some retinal vessels, to semiquantitatively assess this phenotype, the number of affected vessels in each image was counted by an observer blinded to age and genotype.

### Electroretinography

Electroretinography (ERG) measures the electrical responses of various cell types in the retina, including the photoreceptors, inner retinal cells (bipolar and amacrine cells), and the ganglion cells, which are sensitive to hypoxia and structural damage to the neural retina. To assess functional consequences of accelerated microvascular aging associated with IGF-1 deficiency, full-field ERG measurements were performed in the experimental mice as described by Chakraborty et al. (2020). Following dark adaptation, mice were anesthetized with ketamine/xylazine and eyes were dilated using 1% cyclopentolate eye drops. ERGs were recorded with the Diagnosys Espion E3 ERG system (Diagnosys LLC, Lowell, MA, United States). Scotopic ERGs were recorded with a strobe flash stimulus of 157 cd-s m^–2^ presented to the dark-adapted mouse followed by light-adaptation for 5 min at 29.03 cd m^–2^. Photopic responses were recorded from 25 averaged flashes at 77 cd-s m^–2^ for white light. Flicker ERGs were recorded for 30 s in response to a 10 Hz flicker stimulus.

### Neurovascular Coupling

Neurovascular coupling responses were tested in a subset of 2 year-old and young control mice (∼3–6 months of age) according to our previously reported protocol (Tarantini et al., 2017a,^2018^; Yabluchanskiy et al., 2020). Mice were anesthetized with isoflurane (4% for induction and 1–2% for maintenance during the surgery and measurements). A femoral artery cannula was placed in each animal to monitor and maintain blood pressure in the physiological range during the procedure (between 90 and 110 mmHg). Mice were then endotracheally intubated and ventilated (MousVent G500; Kent Scientific Co., Torrington, CT, United States). Rectal temperature was maintained at 37°C using a thermostatic heating pad (Kent Scientific Co., Torrington, CT, United States). Mice were placed in a stereotaxic frame (Leica Microsystems, Buffalo Grove, IL, United States) and a thinned-skull cranial window was prepared. To prepare the thinned-skull window, the scalp and periosteum were resected and the skull was thinned with a sterile scalpel blade. Nitrocellulose lacquer was then applied to the surface of the skull to allow for appropriate optics and light spreading. A laser speckle imager (Perimed, Järfälla, Sweden) was positioned 10 cm above the window. The change in CBF in response to whisker stimulation was measured by stimulating the whiskers on one side of the mouse for 30 s intervals and measuring the change in CBF of the contralateral whisker barrel cortex. The change in CBF is expressed as the percent increase from the baseline.

### Enzyme-Linked Immunosorbent Assay

Blood was collected via puncture of the submandibular vein with a sterile lancet or 25G needle. The whole blood sample was allowed to coagulate for 20 min at room temperature and was then centrifuged at 2,500 × *g* for 20 min at 4°C. Serum was collected and stored at –80°C until use. IGF-1 concentration in the serum samples was measured by enzyme-linked immunosorbent assay (ELISA) (R&D Systems, Minneapolis, MN, United States) as previously reported (Toth et al., 2014). An IGF-1 control sample was included on each plate. Serum IGF-1 levels were reported in ng mL^–1^.

### Immunofluorescence

Whole eyes were collected from transcardially perfused mice and fixed in EM grade 4% paraformaldehyde (PFA) for 4 h at 4°C. Eyes were paraffin-embedded and sectioned at 6 μm thickness onto glass slides. After deparaffinization, slides underwent antigen retrieval in a solution of 10 mM citrate buffer (pH 6.0) for 20 min in a vegetable steamer and were then allowed to cool for 20 min at room temperature. Slides were then pre-treated with 1% sodium borohydride for 90 s, followed by three water washes and three 1× PBS washes. Blocking was performed in a blocking solution containing 5% BSA, 1% fish gelatin, 2% donkey serum, and 0.5% Triton in 1× PBS for 1 h at room temperature, followed by incubation in antibody overnight at 4°C in a humidity chamber (antibodies are listed in Table 1). The slides were then washed four times in 1× PBS for 10 min, incubated in secondary antibodies at a concentration of 1:500 for 1–2 h at room temperature, washed four additional times in 1× PBS, and then mounted with Prolong Diamond with 4’,6-diamidino-2-phenylindole (DAPI) (Thermo Fisher Scientific, Waltham, MA, United States) and coverslipped. An Olympus BX62 microscope with a 20× air, 40× air, or 100× oil objective (Olympus Life Science, Waltham, MA, United States) was used for fluorescent imaging. To semiquantitatively assess the presence of GFAP labeling of gliosis, each image was scored by an observer blinded to age and genotype. Scores were assigned as follows: 0: no gliosis (GFAP labeling in endfeet only), 1: mild gliosis (very little sign of GFAP penetrating into other retinal layers), 2: moderate/intermittent gliosis, 3: elevated gliosis, 4: very high levels of gliosis.

**TABLE 1:** Antibodies.

Antigen	Species	Use	Source	References
4-HNE	Rbt-PC	IF	Alpha Diagnostics, cat# HNE11-S	Liou and Storz, 2015
Endomucin	Rat-MC	IF	Millipore Sigma, cat# MAB2624, clone V.5C7 RRID:AB_10807039	
GFAP	Ms-MC	IF	Millipore Sigma, cat# G3893-100UL	Wang et al., 2006
α-SMA	Ms-MC	IF	Millipore Sigma, cat# 113200 clone 1A4, RRID:AB_477010	Jackson-Weaver et al., 2020
Desmin	Ms-MC	IF	ThermoFisher, Cat# MS-376-S1 RRID:AB_61166	Boscolo Sesillo et al., 2019
Igf1R	Rbt-MC	IF	Abcam, cat# ab182408	Liu et al., 2018
Cone Arrestin (Arr4)	Rbt-PC	IF	Cheryl Craft, University of Southern California LUMIj-mCAR, RRID:AB_2314753	Brown et al., 2010; Chakraborty et al., 2010
Calbindin	Ckn-PC	IF	Synaptic Systems, Inc. Cat#214 006, RRID:AB_2619903	Rojek et al., 2019
SV2	Ms-MC	IF	Developmental Studies Hybridoma Bank,	Pang et al., 2018
Rhodopsin	Ms-MC	IF	Clone 1D4 generously shared by Muayyad Al-Ubaidi at the University of Houston. Can be purchased from Santa Cruz Biotechnology, cat# sc-57432, RRID:AB_785511	Kakakhel et al., 2020
VGLUT1	GP-PC	IF	Millipore Sigma, AB5905, AB_2301751	
M-opsin	Rbt-PC	IF	Novus Biologicals Cat# 110-74730 (opsin1),	

*Rbt-PC, rabbit polyclonal; Rat-MC, rat monoclonal; Ms-MC, mouse monoclonal; Ckn-PC, chicken polyclonal; GP-PC, guinea pig polyclonal.*

### Histological Analysis of Bleeds

Brains and eyes were collected from transcardially perfused mice (described above) and fixed in 4% paraformaldehyde for 48 or 4 h, respectively, at 4°C. Brains and eyes were stored in PBS at 4°C until they were embedded in paraffin. Serial coronal brain sections were cut at 8 μm thickness, yielding approximately 1,000 sections per brain. Every twelfth slide from each brain was stained with 3,3-diaminobenzidine (DAB) (Vector Laboratories/Maravai LifeSciences, San Diego, CA, United States) to reveal hemorrhages, and counterstained with Gill’s No. 1 hematoxylin (Millipore Sigma, St. Louis, MO, United States) to show brain structure. DAB reacts with endogenous peroxidases in red blood cells generating a dark brown product that allows for easy and precise detection of extravasated blood in the brain parenchyma. All stained sections were imaged at 10× (for brains) or 20× (for retinas) using the VS120-L100-W Virtual Slide Microscope (Olympus Life Science, Waltham, MA, United States). Images were then analyzed by an observer blinded to genotype, group, and age. ImageJ 1.52p (NIH, Bethesda, MD, United States) software was used to quantify and measure the size of hemorrhages. Images were color deconvolved and thresholded uniformly; then, the pixel intensity integrated density was measured on the selected bleed area using a protocol and ImageJ macro described in (Nyúl-Tóth et al., 2020). Identified hemorrhages were then mapped to specific brain regions by comparing images to the *Allen Mouse Brain Atlas.*^2^ A similar workflow was followed for identifying microbleeds in eyes. Eyes were serially sectioned at 6 μm thickness, yielding approximately 200 sections per eye. Every sixth section was stained with hematoxylin and DAB. A brain section adjacent to one with a bleed was included in each batch of retinal staining to serve as a positive control for the DAB. However, no microbleeds were identified in the eye sections so bleeds could not be counted, measured, or mapped to regions of the eye.

### Morphometry

Central retinal sections (through the optic nerve) were stained with hematoxylin and eosin (H&E), and then imaged at 20× on a VS120-L100-W Virtual Slide Microscope. Spidergrams were generated by measuring outer nuclear layer (ONL), outer plexiform layer (OPL), and inner nuclear layer (INL) thickness at defined distances from the optic nerve head. Values were captured from three sections per eye, by an observer blinded to age and genotype and at least three eyes per genotype/age/group were measured. Thickness measurements were made using Adobe Photoshop CS6.

### Statistical Analysis

Statistical analyses were performed using Graphpad Prism version 9.2.0. Differences between groups were analyzed by two-tailed unpaired *t*-tests (to compare two groups), one-way ANOVA with Tukey’s *post hoc* comparison (to compare more than two groups), or two-way ANOVA with Tukey’s *post hoc* comparison (in cases where there were two different variables). Differences between survival curves were assessed by Log-rank (Mantel–Cox) test. When data were not normally distributed, the Mann–Whitney test was used.

## Results

### Insulin-Like Growth Factor 1 Deficiency Exacerbates the Development of Hypertension-Induced Cerebral Microhemorrhages

**FIGURE 1:**
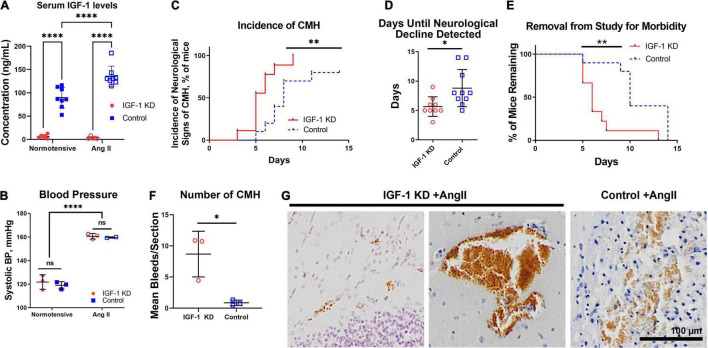
Insulin-like growth factor 1 (IGF-1) deficiency exacerbates the development of hypertension-induced CMH at 1 year of age. **(A–G)** *Igf1^f/f^* mice were treated with TBG-Cre-AAV8 (IGF-1 KD) or TBG-GFP-AAV8 (control) at 4 months of age. At 1 year of age, hypertension was induced with Ang II and L-NAME. **(A)** At 1 year of age, serum ELISAs showed decreased circulating IGF-1 in IGF-1 KD mice (^****^P < 0.0001, by two-way ANOVA with Tukey’s multiple comparisons correction). **(B)** Systolic blood pressures were significantly increased in mice receiving Ang II + L-NAME treatment versus untreated mice, regardless of genotype (^****^P < 0.0001, by two-way ANOVA with Tukey’s multiple comparisons correction). **(C,D)** Hypertensive IGF-1 deficient (IGF-1 KD) mice exhibit neurological signs of CMH earlier than control hypertensive animals. Survival curves plot the day on which neurological score dropped below 18, day 0 is the day the Ang II delivery started [^**^P ≤ 0.01, by Log-rank (Mantel–Cox) test **(C)** and *P < 0.05, by unpaired *t*-test, **(D)**]. **(E)** Hypertensive IGF-1 KD mice were removed from the study and sacrificed when neurological score dropped to 15 or lower [**P ≤ 0.01, by Log-rank (Mantel–Cox) test]. **(F,G)** Bleeds were visualized by staining with DAB, reddish-brown. **(F)** Bleeds were counted in every twelfth section throughout of serially sectioned brains. The number of bleeds per section was averaged across all counted slides for a given mouse. Plotted is mean number of bleeds per section, each symbol reflects one mouse (*N* = 3 mice per group). Shown are mean ± SD (*P < 0.05, by unpaired *t*-test). **(G)** shows representative example bleeds. Scale bar, 100 μm, original magnification 10×.

To study the effects of IGF-1 deficiency on the development of vascular pathologies in the central nervous system, we used a mouse line in which exon 4 of the IGF-1 gene is floxed (*Igf1^f/f^*). We knocked down circulating IGF-1 levels in young adult mice (4 months of age) by injection of an AAV carrying a liver-specific promoter driving Cre recombinase expression [TBG-Cre-AAV8, here referred to as IGF-1 KD (“knockdown”)]. Control animals (also *Igf1^f/f^*) received AAV8-TBG-eGFP at 4 months of age (here referred to as control). The liver is the primary source of paracrine IGF-1 (Ungvari and Csiszar, 2012; Blum et al., 2018), and knocking down IGF-1 production in hepatocytes causes a significant decrease in serum levels of IGF-1. Using ELISAs on serum harvested at 1 year of age, we confirmed that this AAV-mediated approach results in almost complete elimination of circulating IGF-1 levels in our knockdown animals (Figure 1A). We elicited CMHs at 1 year of age by inducing hypertension using a well-established paradigm (Toth et al., 2015b; Tarantini et al., 2017c) wherein Ang II is delivered via osmotic mini-pump and L-NAME is delivered in drinking water (Figure 1B). Normotensive control mice did not receive Ang II or L-NAME. To track the onset of neurological signs of CMHs, mice underwent daily neurological scoring which involved assessment of six different criteria on an 18-point scale. As expected, hypertensive IGF-1 KD mice experienced an earlier onset of neurological signs of CMHs (defined as a drop in neurological score from 18 to 17 or below) compared to control animals (Figures 1C,D). No normotensive animals (either IGF-1 KD or control) exhibited a change in neurological score. Tissues were collected when mice exhibited a neurological score of 15 or lower (or at 28 days after insertion of the mini-pumps). Consistent with their earlier onset of neurological signs of CMHs, hypertensive IGF-1 KD mice were removed from the study and euthanized at a significantly earlier time than control mice due to severe neurological decline (Figure 1E). CMHs were identified histologically by DAB staining (reddish brown color, Figure 1G) which reacts with endogenous peroxidases in red blood cells. We calculated the mean number of bleeds per brain section and found that hypertensive IGF-1 KD exhibited significantly more bleeds per section than hypertensive control animals (Figures 1F,G).

**FIGURE 2:**
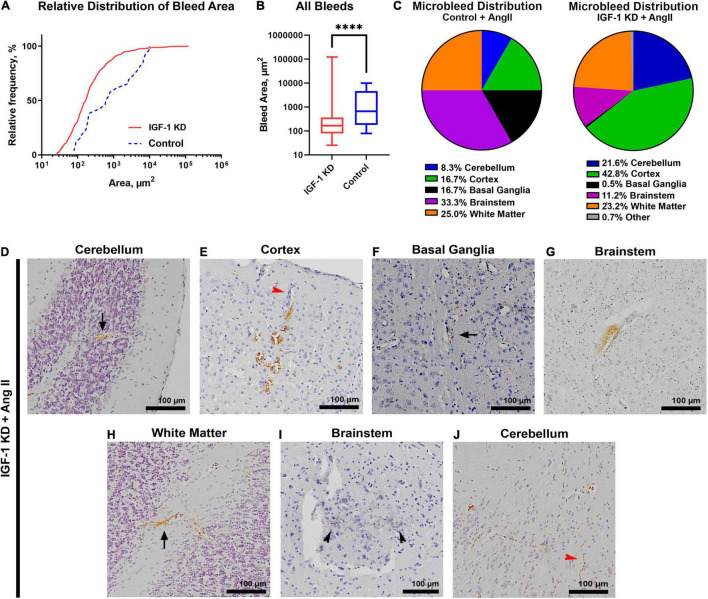
Insulin-like growth factor 1 (IGF-1) deficiency increases the number of hypertension-induced cerebral microhemorrhages (CMHs) at 1 year of age. **(A)** The number of detected brain bleeds (from three control and three IGF-1 KD age-matched, 1-year-old brains) is plotted (relative frequency) as a function of bleed area. IGF-1 KD hypertensive mice have a greater relative frequency of smaller bleeds versus control hypertensive mice. **(B)** The mean bleed size was also smaller in IGF-1 KD mice versus controls (^****^P < 0.0001, Mann Whitney test for non-parametric data). Plotted is the median value, bars represent min/max. **(C)** The pie charts show the bleed locations in hypertensive IGF-1 KD (*N* = 573 bleeds) and control brains (*N* = 12 bleeds). Notably, IGF-1 KD mice had more bleeds in the cerebellum and cortex versus controls. Panels **(D–J)** show representative images of microbleeds in each of the brain regions represented in the pie charts from panel **(C)** (arrows show microbleeds). Black arrowheads denote darker brown 3,3-diaminobenzidine (DAB) staining showing older bleeds. Red arrowheads show bleeds emanating from blood vessels. Scale bars: 100 μm, original magnification 10×. IGF-1 KD: *Igf1^f/f^* + TBG-Cre-AAV8; control: *Igf1^f/f^* + TBG-GFP-AAV8.

To further characterize CMHs, we measured the area of all identified bleeds. The cumulative size distribution of all detected bleeds is plotted in Figure 2B. IGF-1 deficient hypertensive mice had a greater number of bleeds of all sizes than hypertensive control mice but the overall size of bleeds in IGF-1 KD was significantly smaller than in controls (Figures 2A,B). Each identified bleed was mapped to its brain region using the online *Allen Mouse Brain Atlas* (last accessed 10/01/2021).^3^ Each bleed was given a broad identifying region (shown in Figure 2C) as well as a specific brain region. In the hypertensive IGF-1 knockdown animals, the largest fraction of CMHs occurred in the cortex (42%), although CMHs were detected throughout the brain (examples shown in Figures 2D–J, black arrows indicate especially small or hard to see bleeds). In control animals the largest fraction of CMHs occurred in the brainstem and white matter (33 and 25%, respectively), though very few bleeds were detected in control brains overall. Some of the CMHs that occurred can be traced back to an adjacent arteriole or capillary in the brain (Figures 2D–J, red arrowheads indicate vessels). These findings demonstrate that hypertensive IGF-1 knockdown animals have an earlier onset and increased number of CMHs in the brain compared to hypertensive control animals, confirming that IGF-1 deficiency exacerbates microvascular fragility in the mouse brain, mimicking the aging phenotype.

### Animals With Cerebral Bleeds Do Not Exhibit Signs of Retinal Bleeds

**FIGURE 3:**
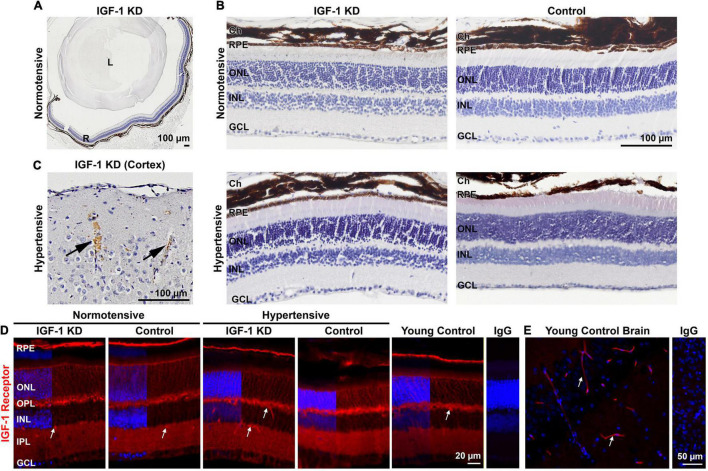
Insulin-like growth factor 1 (IGF-1) deficient hypertensive mice do not show evidence of retinal microbleeds at 1 year of age. **(A,B)** Retinas from 1-year-old hypertensive IGF-1 knockdown (KD) and control animals were stained with hematoxylin and 3,3-diaminobenzidine (DAB). **(C)** Brain sections containing CMH were included as positive staining controls in each batch of retinal sections, 20 × (arrows denote microbleeds). **(D,E)** Retinal **(D)** and brain **(E)** sections were stained for IGF-1 receptor (red) and counterstained with DAPI (blue). *N* = 3 mice per group. Scale bars: 100 μm **(A–C)**, 20 μm **(D)**, 50 μm **(E)**, original magnification 10× **(A,C)**, 20× **(B,E)**, 40× **(D)**. R, retina; L, lens; Ch, choroid; RPE, retinal pigment epithelium; ONL, outer nuclear layer; INL, inner nuclear layer; GCL, ganglion cell layer; OPL, outer plexiform layer; IPL, inner plexiform layer. IGF-1 KD: *Igf1^f/f^* + TBG-Cre-AAV8; control: *Igf1^f/f^* + TBG-GFP-AAV8.

To assess whether mice in which CMHs developed in the brain also exhibited bleeds in the retina, we serially sectioned eyes collected from the same 1-year-old IGF-1 knockdown and control hypertensive and normotensive animals used for the experiments presented in Figures 1, 2. Every 6th retinal section was stained with DAB to detect extravasated red blood cells (RBCs) and counterstained with hematoxylin. Our focus in these studies was identifying bleeds originating from the retinal vasculature. While the choroidal vasculature also supplies nutrients and oxygen to the retina, choroidal vessels are anatomically different from retinal and brain vessels (for example, choriocapillaris vessels are fenestrated and do not participate in the blood–retina barrier). In addition, the pigmented nature of the mouse choroid masks any DAB staining. The DAB stained retinal sections were scored by multiple blinded observers for the presence of any sign of bleed in the retina. However, no evidence of bleeds was detected in any retinal section examined in any region of the retina from any of the groups (example images are shown in Figures 3A,B). To confirm that this was not a staining artifact, brain sections adjacent to those in which cerebral bleeds had previously been detected were included in each retinal staining experiment as positive controls (Figure 3C). To verify that the retina carries receptors for IGF-1, we performed immunofluorescence labeling for IGF-1 receptor (red, Figures 3D,E). IGF-1 receptor is localized throughout the retina, and is particularly enriched in the retinal pigment epithelium (RPE) layer, in the two synaptic layers (inner and outer plexiform layers), and in blood vessels (white arrows). The labeling pattern for IGF-1 receptor is similar in IGF-1 KD and control retinas and in hypertensive and normotensive retinas (Figure 3D). IGF-1 receptor labeling in the brain with pronounced vascular labeling (white arrows) is shown in Figure 3E. These findings suggest that the retina is not as susceptible as the brain to the development of CMHs.

### Circulating Insulin-Like Growth Factor 1 Deficiency Does Not Lead to Retinal Degeneration

**FIGURE 4:**
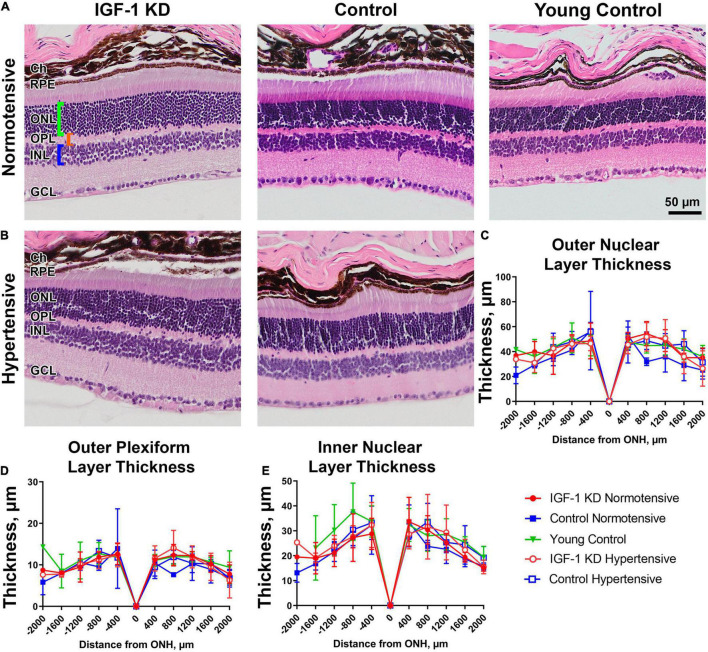
Insulin-like growth factor 1 (IGF-1) deficient mice do not show signs of retinal degeneration at 1 year of age. Panels **(A,B)** shown are H&E stained retinal sections from 1-year-old normotensive/hypertensive IGF-1 KD, age-matched control, and young control mice. Green bracket highlights ONL, orange bracket highlights OPL, blue bracket highlights INL. **(C–E)** Spidergrams show the thickness of the ONL **(C)**, outer plexiform layer **(D)**, and inner nuclear layer **(E)** as measured from the optic nerve head (ONH). Plotted are means ± SD. *N* = 3–5 mice per group. Ch, choroid; RPE, retinal pigment epithelium; ONL, outer nuclear layer; OPL, outer plexiform layer; INL, inner nuclear layer; GCL, ganglion cell layer. Original magnification, 20×. Scale bar: 50 μm.

As part of our general evaluation of the effects of circulating IGF-1 KD in the retina, we also undertook morphometric measurements to assess retinal degeneration (representative images shown in Figures 4A,B). One-year-old IGF-1 knockdown mice (regardless of whether they were hypertensive or normotensive) did not exhibit thinning of the ONL (photoreceptors, Figure 4C), INL (retinal interneurons, Figure 4E), or OPL (the layer where photoreceptor terminals form synapses with bipolar and horizontal cells, Figure 4D) compared to age-matched controls and young (3 month old) controls.

**FIGURE 5:**
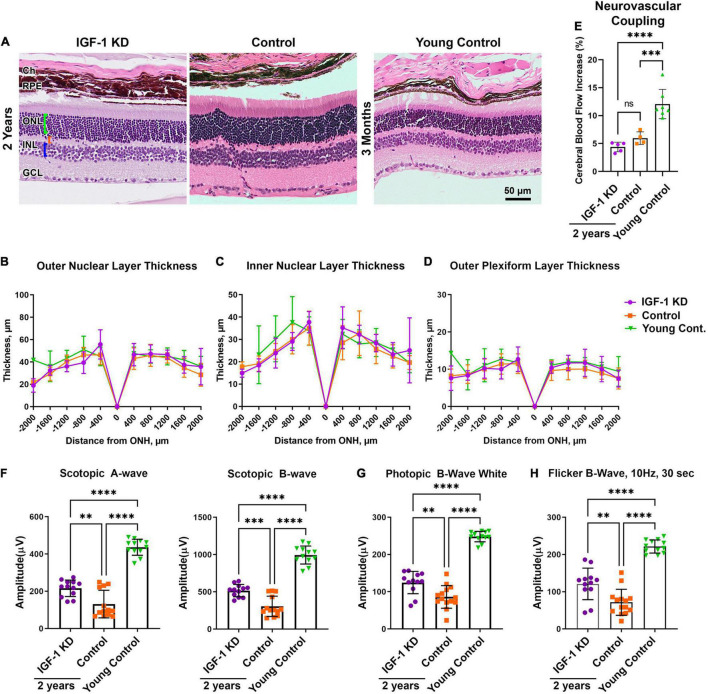
Insulin-like growth factor 1 (IGF-1) deficiency slows age-related declines in retinal function at 2 years of age. **(A–D)** Retinas from 2-year-old IGF-1 KD, age-matched control, and young control animals were H&E stained and the thickness of retinal layers was measured. Green bracket highlights ONL, orange bracket highlights OPL, blue bracket highlights INL. **(B–D)** Spidergrams presenting the thickness of the outer nuclear layer **(B)**, inner nuclear layer **(C)**, and outer plexiform layer **(D)** are shown. Young control group is replotted from Figure 4 as a control. There are no significant differences in thickness between the groups (*N* = 5–8 mice/group). **(E)** Change in blood flow in the somatosensory cortex in response to whisker stimulation was measured in 2-year-old mice as a measure of neurovascular coupling. **(F–H)** Two-year-old animals and controls underwent dark-adapted [**(F)** scotopic], light adapted [**(G)** photopic], or flicker [**(H)** photopic] electroretinography (ERG) to measure retinal function. *N* = 6–8 mice/group, each symbol represents an individual eye. Plotted are means ± SD ^**^P < 0.01, ^***^P < 0.001, ^****^P < 0.0001 by one-way ANOVA with Tukey’s *post hoc* comparison. Ch, choroid; RPE, retinal pigment epithelium; ONL, outer nuclear layer; OPL, outer plexiform layer; INL, inner nuclear layer; GCL, ganglion cell layer. Scale bar: 50 μm. Original magnification, 20×. IGF-1 KD: *Igf1^f/f^* + TBG-Cre-AAV8; control: *Igf1^f/f^* + TBG-GFP-AAV8.

We next undertook a series of experiments designed to evaluate whether circulating IGF-1 deficiency had any general effects on the retina in aged mice (∼2 years of age). Similar to the case at 1 year of age, we observed no signs of retinal degeneration in IGF-1 KD or age-matched controls compared to young control mice (young control group replotted from Figure 4 for ease of comparison) (Figures 5A–D). Incipient photoreceptor degeneration is often preceded by mislocalization of photoreceptor outer segment proteins such as rod and cone opsins, however, we observed no signs of this in 1 or 2-year-old IGF-1 KD animals (or age-matched controls, Supplementary Figure 1). Because retinal function does not correlate directly with retinal degeneration, and it has been well established that there is significant age-related loss of retinal function without significant structural degeneration (Li et al., 2001; Gresh et al., 2003; Samuel et al., 2011; Ferdous et al., 2021), we also conducted scotopic and photopic electroretinography on 2-year-old animals to assess rod and cone function. As expected, 2-year old mice (both IGF-1 knockdown and control) exhibited significant reductions in both dark adapted (rods, Figure 5F) and light-adapted (cones, Figures 5G,H) responses compared to young (3-month-old) controls. These findings are consistent with prior studies in C57BL/6 showing significant age-related loss in rod and cone function with little or no photoreceptor degeneration (Li et al., 2001; Gresh et al., 2003; Samuel et al., 2011; Ferdous et al., 2021). IGF-1 KD mice were partially protected from age-related decreases in rod and cone function compared to control mice (Figures 5F–H), exhibiting scotopic a- and b- wave amplitudes and photopic b-wave amplitudes that were slightly higher than those in age-matched controls.

These data were in contrast to findings in the brain, wherein circulating IGF-1 deficiency led to signs of neuronal dysfunction such as gait defects and cognitive decline as a result of impaired neurovascular coupling when assessed at 1 year of age (Toth et al., 2014; Tarantini et al., 2017c). Thus, we asked whether neurovascular coupling in the brain was also impaired in IGF-1 KD animals at 2 years of age. Measurements of CBF changes in the somatosensory cortex in response to whisker stimulation were performed on aged 2-year-old IGF-1 KD animals, age-matched controls, and young (3–6 month) controls. Both groups of 2-year-old mice exhibited significantly reduced neurovascular coupling compared to young mice (Figure 5E). IGF-1 KD mice had mean values slightly less than age-matched controls, however the difference did not achieve statistical significance. These data are consistent with the view that genetic IGF-1 deficiency at younger ages promotes accelerated cerebromicrovascular aging, which mimics the effects of age-related decline in circulating IGF-1 manifested at later ages in wild type mice.

**FIGURE 6:**
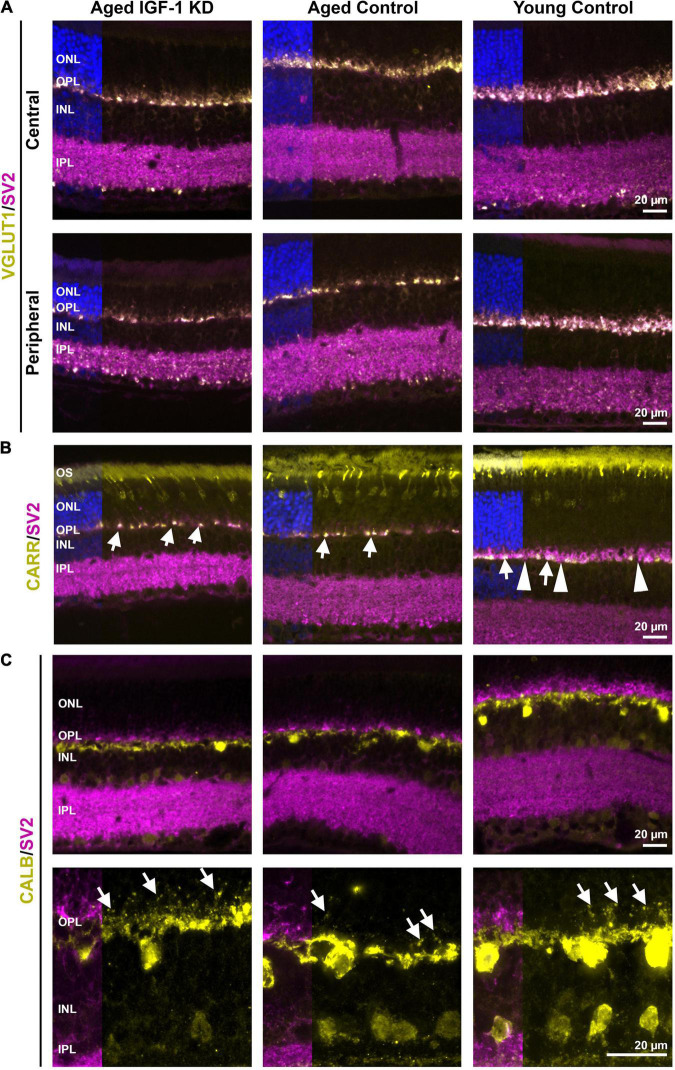
Circulating insulin-like growth factor 1 (IGF-1) deficiency does not lead to gross synaptic abnormalities at 2 years of age. Retinal sections from the indicated groups were collected at 2 years of age (left and center) or at 3–6 months of age (right). Sections were labeled for VGLUT1 [yellow, **(A)**], cone arrestin [CARR, yellow, **(B)**], or calbindin [CALB, yellow, **(C)**] and SV2 [magenta, **(A–C)**]. Sections were counterstained with DAPI (blue). Panel **(A)** shown are representative images from both the central and peripheral retina. **(B)** Arrows highlight example cone pedicles co-labeled for CARR and SV2. Arrowheads highlight example rod spherules (SV2 positive, CARR negative). **(C)** Arrows highlight horizontal cell projections in the outer plexiform layer. Scale bars: 20 μm, original magnification 40× [**(A–C)**-top], and 100× [**(C)**-bottom]. *N* = 3–5 eyes per group. OPL, outer plexiform layer; INL, inner nuclear layer; IPL, inner plexiform layer; ONL, outer nuclear layer; OS, outer segment layer. IGF-1 KD: *Igf1^f/f^* + TBG-Cre-AAV8; control: *Igf1^f/f^* + TBG-GFP-AAV8.

Global IGF-1 knockout retinas exhibit loss of synapses in the OPL. To evaluate whether there are retinal synaptic defects in mice with adult-onset circulating IGF-1 deficiency, we labeled retinas from IGF-1 KD and control animals for VGLUT1 (photoreceptor and bipolar cell terminals) and SV2 (all presynaptic terminals, Figure 6A, magenta). Aged (2-year-old) IGF-1 KD and WT control animals exhibited a thinner layer of photoreceptor terminals in the OPL compared to young control mice, a phenotype that was more pronounced in the peripheral retina than in the central retina (Figure 6A). The OPL contains a mix of rod spherules and cone pedicles, but the terminals we observed in the 2-year-old animals exhibited morphological signs of cone pedicles. To verify this, we co-labeled retinal sections with the cone marker cone arrestin (CARR, yellow, Figure 6B) and SV2 (magenta, Figure 6B). In young control mice, cone terminals (white terminals are co-labeled with SV2/VGLUT1, and white arrows) and rod terminals (magenta label only, arrowheads highlight examples) are present, but the majority of terminals in aged retinas are co-labeled for CARR and SV2 indicating they originate from cones. To help evaluate whether second-order neurons were present at these synapses, we co-labeled retinal sections for SV2 (magenta, Figure 6C) and the horizontal cell marker calbindin (yellow, Figure 6C). Examination of high magnification images showed that horizontal cell process were properly present in 2 year old IGF-1 KD and control mice (Figure 6C bottom, arrows). We performed similar analyses on 1-year-old IGF-1 KD and control animals (Supplementary Figure 2) but found no abnormalities. Combined, these data suggest that circulating IGF-1 deficiency does not exacerbate age-related defects in retinal structure or function.

### Circulating Insulin-Like Growth Factor 1 Deficiency Leads to Signs of Vascular Abnormalities and Gliosis in the Retina

**FIGURE 7:**
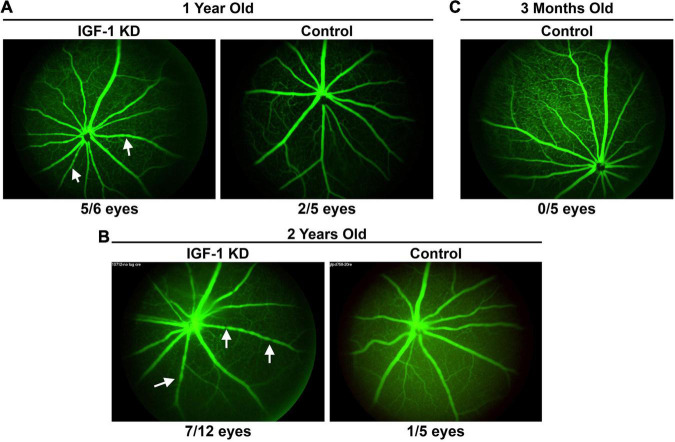
Insulin-like growth factor 1 (IGF-1) deficient mice exhibit vascular abnormalities in the retina. **(A–C)** Fluorescein angiograms were performed in 1– and 2-year-old IGF-1 KD and control animals. Arrows highlight example vessels exhibiting a pattern of vascular constriction (“sausages-on-a-string” phenotype), numbers underneath denote the number of eyes/total eyes that exhibit at least one vessel with the pattern of constriction. IGF-1 KD: *Igf1^f/f^* + TBG-Cre-AAV8; control: *Igf1^f/f^* + TBG-GFP-AAV8.

To further analyze retinal phenotypes associated with circulating IGF-1 deficiency, we performed *in vivo* fluorescein angiography on both IGF-1 KD and control mice (all normotensive) at 1 and 2 years of age. On fluorescein angiograms, we frequently observed patterns of repeated vascular constriction, referred to as “sausage on a string” phenotype (Jacobsen et al., 2002) in one or more retinal vessels in IGF-1 KD mice (Figures 7A,B, arrows). At 1 year of age, 5/6 IGF-1 KD, and only 2/5 age-matched controls exhibited this phenotype. This phenomenon persisted at 2 years of age: 7/12 IGF-1 KD mice had the severe constriction phenotype while only 1/5 age matched controls had the phenotype (Figure 7B). Young control animals did not exhibit this phenotype (Figure 7C).

**FIGURE 8:**
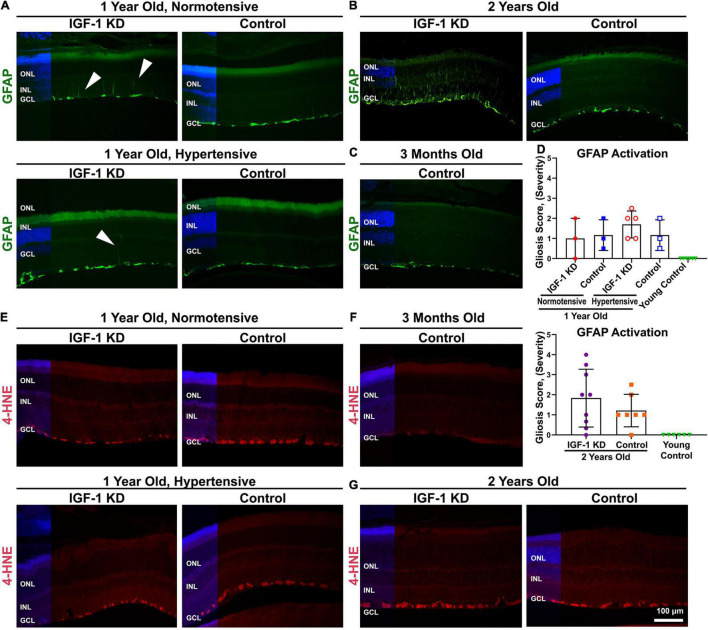
Insulin-like growth factor 1 (IGF-1) deficient mice exhibit signs of reactive gliosis in the retina. **(A–D)** Retinal sections from 1– and 2-year-old IGF-1 KD were labeled for glial fibrillary acidic protein (GFAP) expression (green) and counterstained with DAPI (blue). Arrows highlight incipient Müller cell activation. **(D)** Müller cell activation (upregulation of GFAP, with penetration of labeling outside endfeet) was scored by a blinded observer on a 4-point scale (4 = severe gliosis, 0 = no gliosis). *N* = 3–9 eyes/group. **(E–G)** Oxidative stress was evaluated by labeling retinal sections with 4-HNE (red), a marker of lipid oxidative stress, and counterstained with DAPI (blue). *N* = 3–5 eyes/group. Scale bars: 100 μm. Original magnification, 20×. ONL, outer nuclear layer; INL, inner nuclear layer; GCL, ganglion cell layer. IGF-1 KD: *Igf1^f/f^* + TBG-Cre-AAV8; control: *Igf1^f/f^* + TBG-GFP-AAV8.

We proceeded to evaluate other signs of stress in the retina. We used immunofluorescence to evaluate the expression of retinal glial fibrillary acidic protein (GFAP), an intermediate filament protein normally restricted to the endfeet of Müller glial cells (Figures 8A–D). Increased expression of GFAP leads to staining along the Müller cell body toward the outer layers of the retina, and is a marker of gliosis and retinal stress. At 1 year of age, we observed some minor signs of glial cell activation in IGF-1 KD retinas (Figure 8A, arrowheads). This phenomenon was more pronounced at 2 years of age, wherein significantly increased GFAP labeling was frequently observed in IGF-1 KD retinas compared to age-matched control retinas (Figure 8B). We next scored GFAP-labeled retinas on a scale of 0–4 (0 = no gliosis, 4 = severe gliosis) (Figure 8D). At 1 year of age, gliosis was minor or not detected in all groups. At 2 years of age, there is a high degree of eye-to-eye variability, but even so, IGF-1 KD eyes had a higher median gliosis score than age-matched controls, and 3/9 IGF-1 KD eyes exhibited a high degree of gliosis (score 3 or higher) compared to 0/7 control eyes. To ask whether this gliosis was accompanied by increased oxidative stress, we labeled retinas with a marker of lipid peroxidative stress, 4-hydroxynonenal (4-HNE). However, 4-HNE expression was similar in IGF-1 KD and control mice at 1 and 2 years of age (Figures 8E,G), though staining was modestly increased in aged retinas compared to young controls (Figure 8F). These findings indicate that circulating IGF-1 deficiency leads to abnormalities in the retinal vasculature and retinal gliosis.

## Discussion

Here we confirm that the effects of adult-onset circulating IGF-1 deficiency phenocopy important aspects of cerebrovascular aging (Toth et al., 2015b; Tarantini et al., 2017c) and demonstrate that increased susceptibility to CMHs in IGF-1 deficient mice is associated with imaging signs of vascular defects in the retina. There has been significant research interest in using the eye to model or predict disease in the brain, and several neurodegenerative or cerebrovascular diseases have measurable ocular manifestations (Czako et al., 2020; Istvan et al., 2021). Previous human studies also provide *prima facie* evidence that retinal microvascular changes (microaneurysms and retinal hemorrhage) together with other imaging and histological signs [Müller cell gliosis, retinal nerve fiber layer (RNFL) thinning] predict a higher risk of subsequent stroke in humans (Wong et al., 2001a,b; London et al., 2013; Zhao et al., 2020). Cerebral microhemorrhages are common in the aging human population and predict cognitive decline (Poels et al., 2012; Akoudad et al., 2016) as well as subsequent ischemic and hemorrhagic stroke (Poels et al., 2011). There is strong evidence that circulating IGF-1 deficiency is causally linked to the genesis of CMHs and larger intracerebral hemorrhages (Tarantini et al., 2017c). However, in spite of the shared anatomical origins and important functional and structural similarities between the retinal and cerebral microvasculature (Tata et al., 2015), we found that IGF-1 deficiency did not exacerbate hypertension-induced microbleeds in the retina.

Retinal hemorrhages can occur as part of a wide variety of retinal diseases and conditions, including diabetic retinopathy (Crabtree and Chang, 2021; Jena and Tripathy, 2021), age-related macular degeneration (Avery et al., 1996; Oh et al., 2009; Poels et al., 2012), head trauma in infants (D’Aloisio et al., 2020), and many others, but there is no systematic evidence for age-related retinal microhemorrhages paralleling those seen in the brain. It is not clear whether this is a physiological difference between the retina and the brain or a function of available tools and/or patient populations. How retinal hemorrhages are labeled in human studies may also contribute to a lack of clarity regarding the clinical presence of retinal hemorrhages in aging. A broad spectrum of retinal microvascular changes including retinal hemorrhages, microaneurysms, cotton wool spots, macular edema, other exudates and optic disc swelling are often all grouped under the category of “retinopathy” (Wong et al., 2001b), and it can be challenging to refine individual microvascular manifestations. One of the few studies to specifically evaluate retinal microhemorrhages evaluated patients with cerebral amyloid angiopathy (CAA), a form of vascular dementia in which CMHs are common, found that CAA patients exhibited increased prevalence of retinal microhemorrhages compared to controls, and that there was a correlation between the presence of retinal microbleeds and cerebral bleeds (Alber et al., 2021). Further evaluation into the presence of retinal microhemorrhages in aging, and the extent to which these correlate with age-related CMH would be of great future interest.

There is an emerging body of literature suggesting that retinal microvascular rarefaction, typically measured by a decrease in retinal fractal dimension (a representation of microvascular network complexity) is associated with aging, cerebral microbleeds, cerebral small vessel disease, and the development of cognitive impairment (Hilal et al., 2014; Ong et al., 2014; Chua et al., 2020), although not all studies have confirmed these associations (O’Neill et al., 2021). We did not perform retinal microvascular imaging here, but we did evaluate larger retinal vessels via fundus angiography. Aged IGF-1 deficient animals exhibited worsened patterns of vessel narrowing than control animals. This pattern is similar to the previously described “sausage-on-a-string” phenotype (so named because the constricted vessels resemble a chain of sausage links) and is thought to show regions of vascular instability (Jacobsen et al., 2002). Our “sausage-on-a-string” vessels also bear a resemblance to arteriovenous nicking and focal arteriolar narrowing, defects affecting larger retinal vessel in patients. There is clear evidence that cardiovascular risk factors such as hypertension are associated with changes in retinal microvessels (retinopathy) as well as these larger vessel changes [reviewed in Wong et al. (2001b)]. Combined, our findings suggest that while retinal microhemorrhages are not a part of the hypertensive response in mice with circulating IGF-1 deficiency, other vascular degenerative changes do occur in the retina, and are consistent with the presence of known cerebrovascular defects in IGF-1 knockdown models.

Insulin-like growth factor 1 in the retina in general has been widely evaluated, and as in the brain, the role of IGF-1 as either a protective pro-survival factor or a pro-inflammatory factor depends on the disease context. IGF-1 is an important pro-survival signal for photoreceptors and protects photoreceptors from apoptosis in the context of retinitis pigmentosa (RP) (Arroba et al., 2009; Arroba et al., 2018). IGF-1 is also known to be proangiogenic in eye pathologies associated with angiogenesis such as proliferative diabetic retinopathy (PDR) and wet age-related macular degeneration (AMD) (Arroba et al., 2018). In animal models, the findings have been similarly complicated. Similar to our findings from the circulating IGF-1 knockdown, global IGF-1 knockout animals exhibit signs of increased age-associated Müller cell gliosis in the retina but no overt retinal degeneration (Rodriguez-de la Rosa et al., 2012; Arroba et al., 2016). However, global IGF-1 knockout mice also exhibited significant decreases in rod and cone ERG function by 1 year of age. This is in contrast to our findings demonstrating that adult-onset circulating IGF-1 deficiency led not to declines in ERG function compared to control animals, but rather to a slight retardation of age-related loss of cone and rod function. One possible explanation for this counterintuitive effect is that cells resident to the retina may produce IGF-1 to compensate for any changes caused by circulating deficiency. IGF-1 is produced locally in the retina by multiple cell types including cones (Cao et al., 2001; Zygar et al., 2005; Lofqvist et al., 2009), and may therefore play a key role protecting the retina from circulating IGF-1 deficiency. Support for differential roles for circulating vs. locally-produced IGF-1 in the retina comes from studies utilizing transgenic mice that chronically overexpress IGF-1 in the retina (without increase in circulating IGF-1). Mice with chronic overexpression of intraocular IGF-1 exhibited significant impairments in the blood retinal barrier and loss of tight-junctional integrity, while mice with elevated circulating IGF-1 did not exhibit these phenotypes (Haurigot et al., 2009). However, these intraocular IGF-1 overexpressers also exhibited retinal degeneration, gliosis, and decreases in rod and cone function (Ruberte et al., 2004; Villacampa et al., 2013) similar to the global IGF-1 knockdowns, indicating that the role of IGF-1 in the retina is complex and levels are finely tuned. An additional layer of complexity arises from the fact that both the transgenic overexpression model and the global knockout model have modified IGF-1 levels from birth. Given the key role of IGF-1 in development, such early-onset models may not be the most relevant when studying age-related pathologies associated with IGF-1.

In conclusion, our work highlights the importance of IGF-1 in the maintenance of cerebrovascular and retinal stability and validates adult-onset circulating IGF-1 deficiency as an accelerated aging model. Critically, our findings also show that while the eye can serve as a model for the central nervous system, it does not always mimic every vascular pathology seen in the brain. The role of IGF-1 is complex in both the retina and the brain, but it clearly serves as a vasoprotective factor in both tissues, and further work to understand ways that retinal vascular changes can be used as biomarkers for cerebrovascular changes is urgently needed.

## Data Availability Statement

The original contributions presented in the study are included in the article/Supplementary Material, further inquiries can be directed to the corresponding author/s.

## Ethics Statement

The animal study was reviewed and approved by Institutional Animal Care and Use Committee, University of Oklahoma Health Sciences Center.

## Author Contributions

LM, AC, ZU, and SC: conceptualization. LM, ST, WS, ME, AY, AC, and ZU: methodology. ÁN-T: software. LM, MJ, TM, EB, MB, and SC: validation. LM, ÁN-T, MJ, TM, MB, and SC: formal analysis. LM, ST, ÁN-T, MJ, TM, EB, MB, AY, and SC: investigation. WS, AC, ZU, and SC: resources. LM: writing—original draft. LM, ÁN-T, MJ, TM, EB, MB, WS, AY, AC, ZU, ME, and SC: writing—review and editing. SC and AC: supervision. LM, ST, ZU, AC, SC, and ME: funding acquisition. All authors contributed to the article and approved the submitted version.

## Conflict of Interest

The authors declare that the research was conducted in the absence of any commercial or financial relationships that could be construed as a potential conflict of interest.

## Publisher’s Note

All claims expressed in this article are solely those of the authors and do not necessarily represent those of their affiliated organizations, or those of the publisher, the editors and the reviewers. Any product that may be evaluated in this article, or claim that may be made by its manufacturer, is not guaranteed or endorsed by the publisher.

## Footnotes

1
http://www.med.upenn.edu/gtp/vectorcore

2
https://mouse.brain-map.org/static/atlas

3
https://mouse.brain-map.org/
