# Supplementary material for: Increased Susceptibility to Cerebral Microhemorrhages Is Associated With Imaging Signs of Microvascular Degeneration in the Retina in an Insulin-Like Growth Factor 1 Deficient Mouse Model of Accelerated Aging
Source: Front Aging Neurosci. 2022 Mar 9;14:788296. doi: 10.3389/fnagi.2022.788296 (PMC8959924; doi:10.3389/fnagi.2022.788296)
Supplement: Supplementary file 1 [file Data_Sheet_1.PDF]

Miller et al., Supplementary Material

1.1 Supplementary Figures

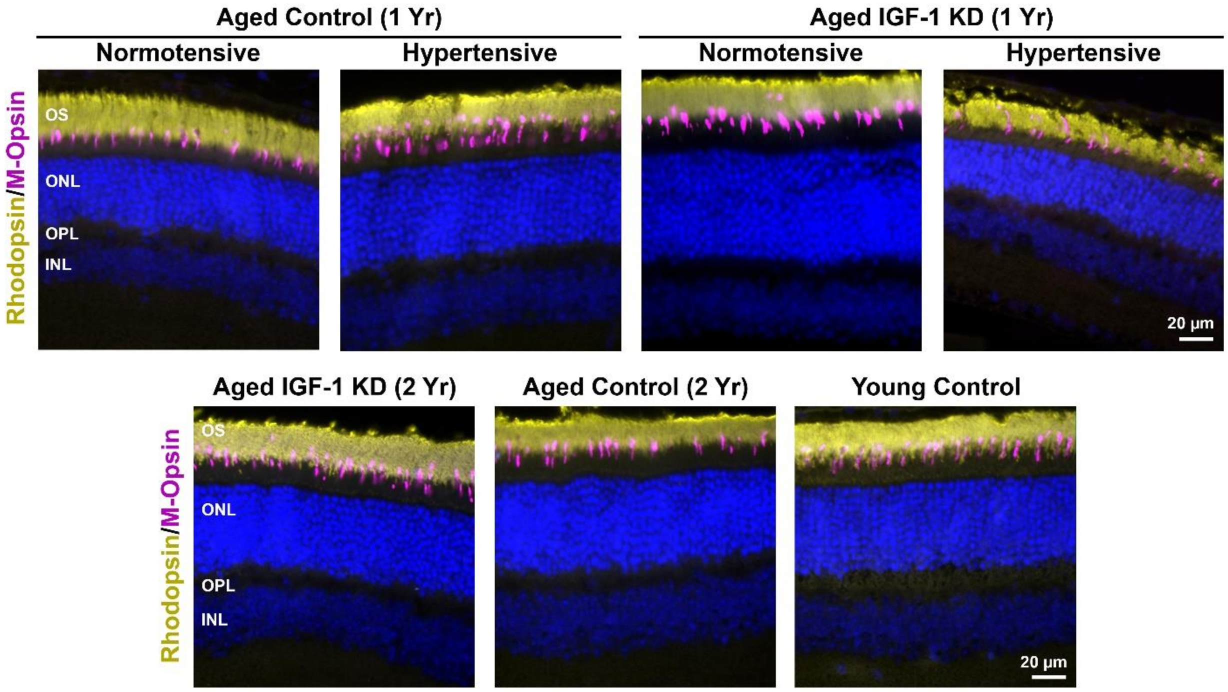

Supplementary Figure 1. Circulating IGF-1 deficiency does not lead to mislocalization of outer segment proteins. Retinal sections from the indicated groups were collected at one year of age and two years of age and labeled for rhodopsin (yellow), M-opsin (cone opsin, magenta) and counterstained with DAPI (blue). As expected, labeling is restricted to the OS layer. Scale bars: 20 µm, original magnification 40x. N=3-5 eyes per group. OPL: outer plexiform layer, INL: inner nuclear layer, IPL: inner plexiform layer, ONL: outer nuclear layer, OS: outer segment layer. IGF-1 KD: Igf1$^{f/f}$ +TBG-Cre-AAV8; control: Igf1$^{f/f}$ +TBG-GFP-AAV8.

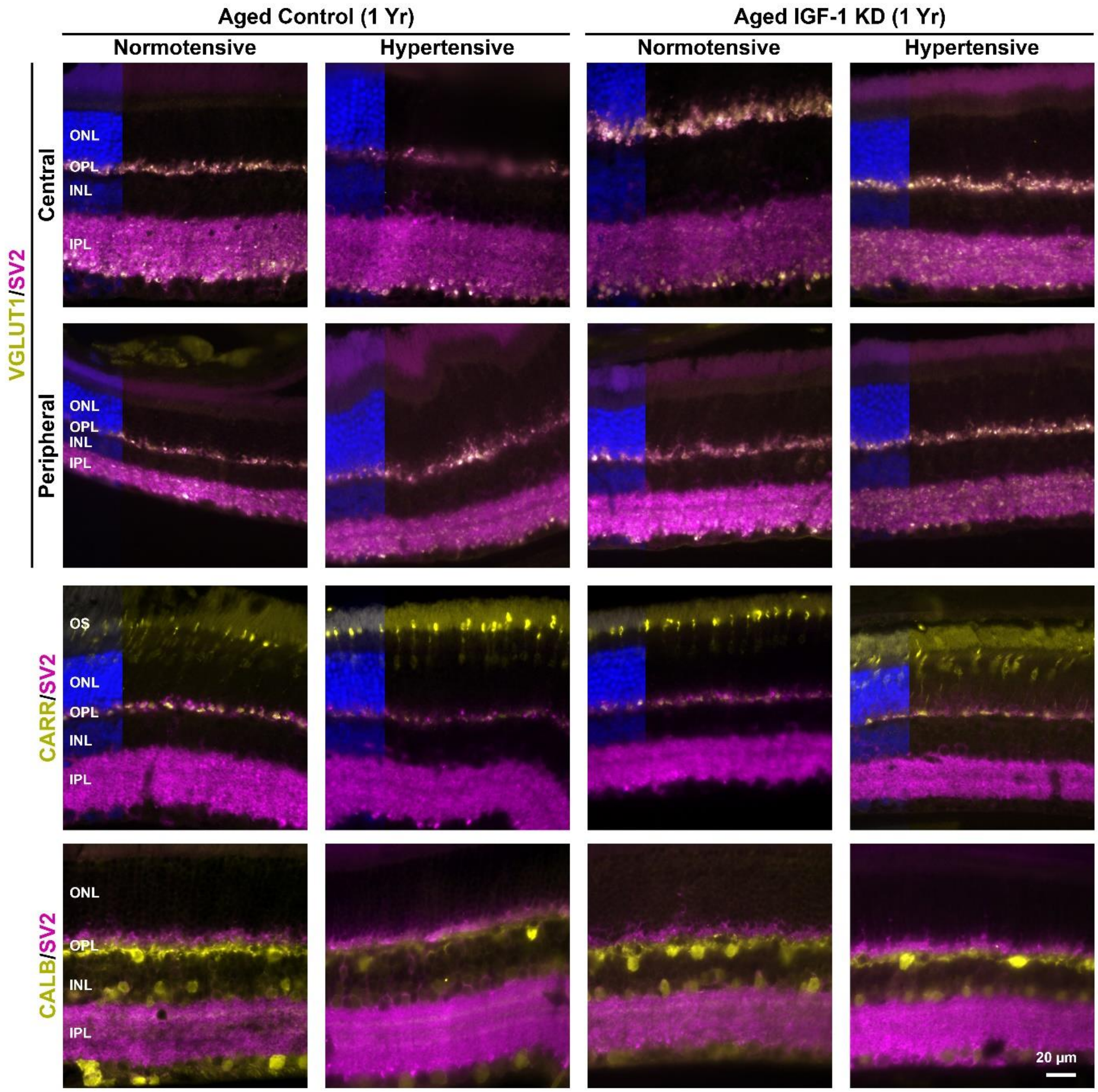

Supplementary Figure 2. Circulating IGF-1 deficiency does not lead to gross synaptic abnormalities. Retinal sections from the indicated groups were collected at one year of age. Sections were labeled for VGLUT1 (yellow, **A**), cone arrestin (CARR, yellow, **B**), or calbindin (CALB, yellow, **C**) and SV2 (magenta, **A-C**). Sections were counterstained with DAPI (blue). **A.** Shown are representative images from both the central and peripheral retina. Scale bars: 20 µm, original magnification 40x. N=3 eyes per group. OPL: outer plexiform layer, INL: inner nuclear layer, IPL: inner plexiform layer, ONL: outer nuclear layer, OS: outer segment layer. IGF-1 KD: Igf1$^{f/f}$ +TBG-Cre-AAV8; control: Igf1$^{f/f}$ +TBG-GFP-AAV8.
